# Gaseous emissions from brake wear can form secondary particulate matter

**DOI:** 10.1038/s41598-024-74378-5

**Published:** 2024-10-06

**Authors:** Anil Patel, Sneha Aggarwal, Lucas Bard, Olivier Durif, Micol Introna, Ana Teresa Juárez-Facio, Minghui Tu, Karine Elihn, Barbara Nozière, Ulf Olofsson, Sarah S. Steimer

**Affiliations:** 1 Department of Environmental Science, Stockholm University, 11418 Stockholm, Sweden; 2 Bolin Centre for Climate Research, 11418 Stockholm, Sweden; 3 Department of Machine Design, KTH Royal Institute of Technology, 10044 Stockholm, Sweden; 4 Department of Chemistry, KTH Royal Institute of Technology, 10044 Stockholm, Sweden; 5 Present Address: Department of Atmospheric and Oceanic Sciences, University of California at Los Angeles, Los Angeles, CA 90095-1565 USA

**Keywords:** Brake wear, Non-exhaust emissions, Secondary particle formation, Air quality, Oxidation flow reactor, Atmospheric chemistry, Atmospheric chemistry

## Abstract

Road traffic is an important source of urban air pollutants. Due to increasingly strict controls of exhaust emissions from road traffic, their contribution to the total emissions has strongly decreased over time in high-income countries. In contrast, non-exhaust emissions from road vehicles are not yet legislated and now make up the major proportion of road traffic emissions in many countries. Brake wear, which occurs due to friction between brake linings and their rotating counterpart, is one of the main non-exhaust sources contributing to particle emissions. Since the focus of brake wear emission has largely been on particulate pollutants, little is currently known about gaseous emissions such as volatile organic compounds from braking and their fate in the atmosphere. This study investigates the oxidative ageing of gaseous brake wear emissions generated with a pin-on-disc tribometer, using an oxidation flow reactor. The results demonstrate, for the first time, that the photooxidation of gaseous brake wear emissions can lead to formation of secondary particulate matter, which could amplify the environmental impact of brake wear emissions.

## Introduction

It is well established that exposure to air pollution can lead to negative effects on respiratory and cardiovascular health. In addition, recent evidence points towards potential negative effects on metabolic function, the central nervous system, pregnancy and developmental outcomes as well as psychiatric conditions^1^. According to global assessments, between 4 and 9 million annual premature deaths as well as hundreds of millions of lost years of healthy life can be attributed to the effects of ambient air pollution^2^.

Road traffic represents a major source of urban air pollutants, contributing through both exhaust and non-exhaust emissions. Due to increasingly strict regulatory standards, exhaust emissions from road vehicles have substantially decreased in high-income countries over the last years. However, non-exhaust emissions are currently unregulated and therefore now exceed exhaust emissions in many countries^3,4^. For this reason, it can be expected that future regulations will include this important source of emissions, as for example suggested in the European Commission proposal for the new Euro7 vehicle standards^5^. One important type of non-exhaust emissions is brake wear particles, which are formed from friction between brake linings and their rotating counterpart. The European Environment Agency (EEA) applies a PM_10_ emission factor for brake wear emissions from light duty vehicles^6^ of 7.5 mg km^−1^ veh^−1^, whereas a recent round robin^7^ found values ranging between 6 and 27 mg km^−1^ veh^−1^. This is in line with or even exceeds the exhaust emission factors for modern light duty vehicles^6^. A similar pattern can be observed for particle number emissions. Recent studies^8–10^ presented particle number emission factors around 1 × 10^11^ to 1.5 × 10^11^ km^−1^ veh^−1^. These emission factors are in the same order of magnitude as those for exhaust of modern gasoline engines equipped with particulate filters, which are typically in the range 1 × 10^11^ to 1 × 10^12^ km^−1^ veh^−1^^11^.

Particulate emissions have been extensively studied due to their adverse effects on human health. In contrast, only little is currently known about the gaseous emissions from braking and their potential effect on human health and the environment.

It has been demonstrated in previous studies that disc brake temperature strongly affects the number of ultrafine particles emitted from braking^12^; the number of ultrafine particles increases dramatically once the transition temperature is exceeded. Typical values of the transition temperature range from 140 to 240 °C^13–15^. A recent study^16^ linked the exceedance of the transition temperature to the formation of semi-volatile emissions by demonstrating that the number concentration of particles < 200 nm decreased by several magnitudes upon removal of the semi-volatile part. Additional evidence of gaseous brake wear emissions comes from several studies that have shown that brake wear particles have a lower carbon content compared to the original brake lining. This discrepancy tends to increase with the severity of the braking process^17–19^, which indicates that under more severe braking conditions, a significant portion of the carbonaceous material becomes volatilized. This formation of VOCs from the braking process is generally attributed to the thermal decomposition of the organic brake pad constituents^20^. Pyrolysis of phenolic resins, which are commonly used as binders in brake pads, has for example been shown to release VOCs such as benzene, toluene and phenol^21,22^. So far, only very few studies have characterised VOC emissions from brake wear^23,24^, with a large fraction of the VOCs remaining unidentified. Not only has the composition of VOC mixtures from brake wear hardly been studied, but their potential to produce secondary organic aerosols by photooxidation has never been investigated before. This is despite the fact that secondary particle formation could lead to an additional impact of brake wear emissions on air quality and climate that is currently not accounted for. This work presents the first study of this kind.

To investigate the gaseous emissions from brake wear and their photooxidative aging, a series of measurement campaigns were performed as part of the EU-project nanoParticle Emissions from the Transport Sector (nPETS). Detailed results obtained on the gaseous emissions will be published elsewhere. The present study focuses specifically on the hypothesis that photooxidation of gaseous emissions from brake wear can form secondary particulate matter (PM).

## Results and discussion

### Formation of secondary particulate matter

**Fig. 1:**
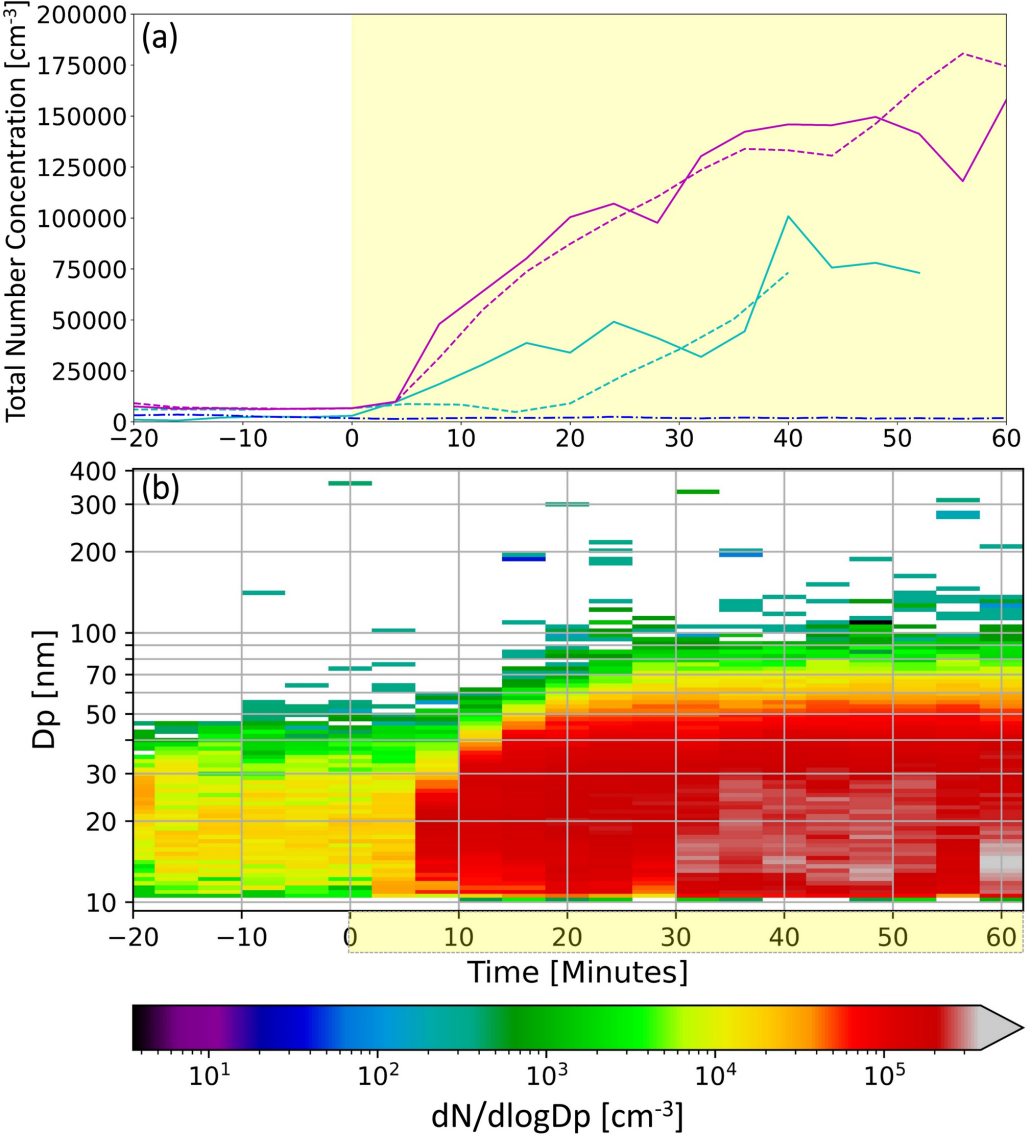
(**a**) Total number concentration in the flow reactor over the tribometer running time. Solid light blue and magenta lines represent the equivalent ambient ages of 7 days (condition I) and 9 days (condition II), respectively, whereas the dashed lines are the repeat for each equivalent age. The dash-dotted dark blue line represents a control experiment where the tribometer was run without a brake pin. (**b**) Corresponding number size distribution over time for an experiment performed at 9 days equivalent age. The times highlighted in yellow indicate the period when the tribometer was turned on (i.e. when wear emissions were generated).

Our results clearly show, for the first time, that photooxidation of gaseous brake wear emissions can lead to the formation of secondary PM (Fig. 1): number concentrations in the oxidation flow reactor simulating atmospheric photochemistry begin to increase once brake wear emissions are starting to be produced by a tribometer. These observed particles are clearly of secondary origin (i.e. formed through chemical reactions) since the brake wear emissions are filtered before being introduced into the flow reactor, removing any primary particles directly emitted from the wear process. Two different conditions were tested that are equivalent to different degrees of photooxidative aging in the atmosphere; condition I (7 days equivalent age) and condition II (9 days equivalent age). Higher particle number concentrations are reached at a higher equivalent ambient age (9 *vs.* 7 days) for the same tribometer running time, i.e. time elapsed since the start of the production of emissions. The background subtracted average number concentrations of newly formed particles at the two different equivalent ages were calculated at 40–52 min tribometer running time to ensure that concentrations had stabilised at both aging conditions. This led to the exclusion of the repeat experiment at 7 days equivalent aging time from this calculation since it only ran to 40 min. The resulting number concentrations are 8.0 ± 1.1 × 10^4^ cm^−3^ at 7 days equivalent ageing (average ± 1σ, one measurement) and 1.4 ± 0.03 × 10^5^ cm^−3^ respective 1.4 ± 0.13 × 10^5^ cm^−3^ at 9 days equivalent ageing (average ± 1σ, measurement I and II) (Fig. 1A). Figure 1B shows development of the size distribution over time for an experiment at 9 days equivalent age. Here one can observe a clear increase in both the number concentration as well as the mode diameter with increasing tribometer running time. A similar trend can be seen for the other experiments, with slight increase in maximum particle sizes at higher equivalent age. A comparison of the development of the number size distributions for all experiments can be found in the supplementary information (Fig. S1). Furthermore, it is interesting to note that it took up to 40 min to reach the maximum particle number concentration for both ageing conditions. The stable particle number concentration after 40 min suggests a stable formation rate of PM after this point. However, this does not necessarily imply stable concentrations of gaseous precursors or a constant gas phase composition throughout the experiment since changes in concentration could be balanced out by changes in PM formation potential with changing precursor composition. Assuming spherical particles and a density of 1.4 g cm^−3^ based on Hallquist et al.^25^, the background subtracted average mass concentrations produced at condition I and II were 2.4 and 2.7 µg m^−3^, respectively at 40–52 min tribometer running time.

### Composition of the freshly emitted organic gases

**Fig. 2:**
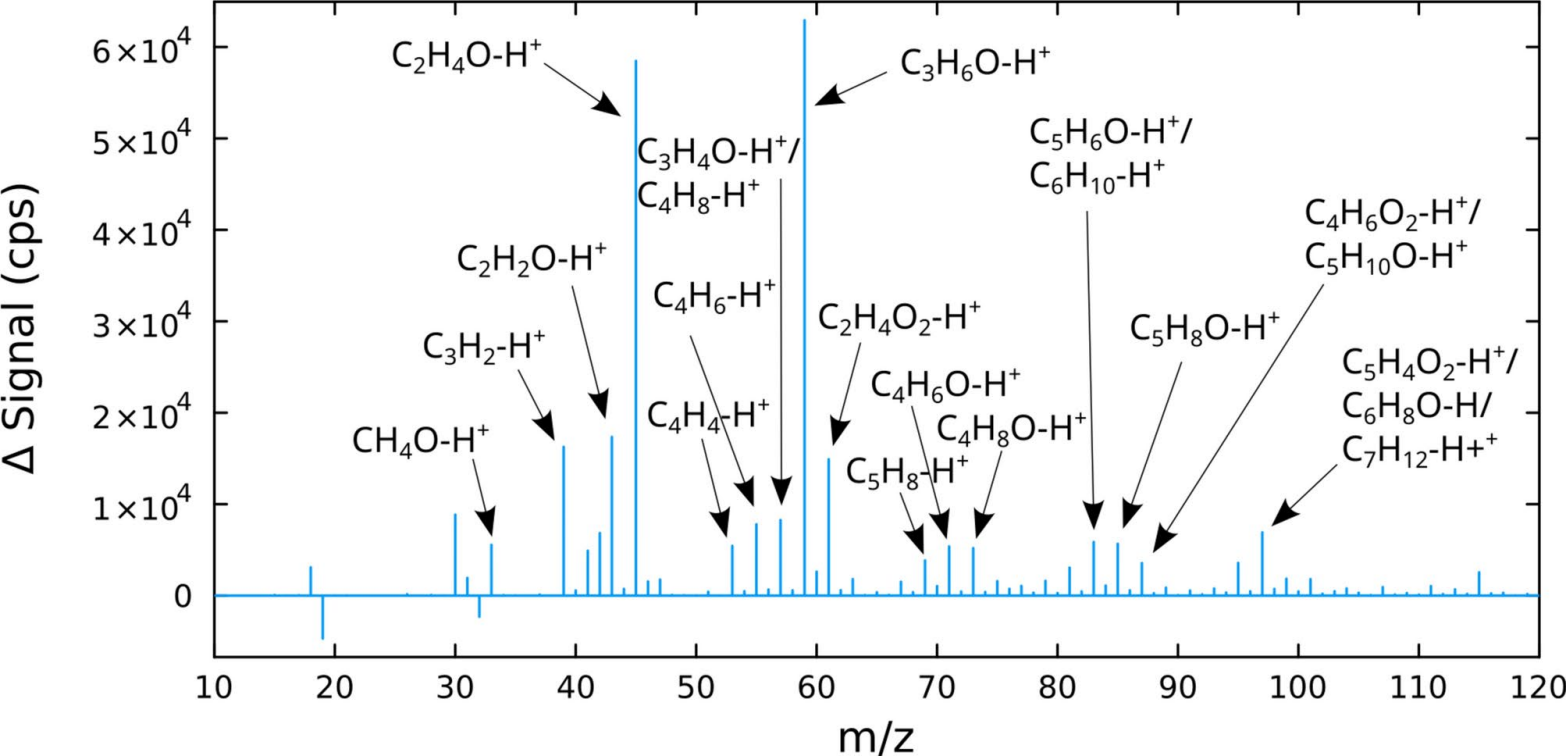
Mass spectrum of gaseous emissions from wear of brake pad material, obtained by on-line mass spectrometry. Assigned molecular formulae are annotated for the most intense peaks. The tribometer was operated at sliding speed = 4 m s^−1^ and contact pressure = 0.9 MPa. To eliminate background noise, the pre-experiment signal was subtracted from the signal recorded 15 min after tribometer start. For enhanced visibility, peak integration was performed for each integer mass (from n − 1/2 to n + 1/2) and the results are presented as a discrete histogram, reconstituting a mass spectrum.

This study included an assessment of the organic gases emitted from the simulated braking process. Masses from 0 to 700 amu were detected in the gas phase using on-line mass spectrometry (Proton-Transfer-Reaction Mass Spectrometer, PTR-MS), with the most intense signals observed between 20 and 150 amu. An example mass spectrum at 15 min tribometer running time is shown in Fig. 2. The mass spectrum reveals a complex chemistry during the friction process, highlighted by the fact that over 150 different mass signals were detected. This exceeded the number of molecules in a previous study of a semi-metallic brake material^23^ utilizing gas chromatography coupled with mass spectrometry (GC–MS), which reported a maximum of 13 different compounds depending on the braking conditions. A more recent study of a ceramic and a semi-metallic brake pad identified 85 individual VOCs (with an additional ∼ 20 tentatively identified) utilizing offline analysis via GC–MS while online analysis with PTR-MS detected 93 individual ions^24^. Differences with the PTR-MS results in our study may reflect the difference in brake materials.

A detailed analysis of the gas phase composition for different tribometer running conditions in comparison with emissions from other brake pad materials will be made in a separate publication. Briefly, the molecules assigned to the detected *m/z* were the organic chemical compounds commonly observed in polluted air. They showed great diversity, including hydrocarbons and their nitrogenated and oxygenated derivatives (occasionally containing up to three oxygen atoms), both saturated and unsaturated, ranging from one to over 10 carbon atoms, and existing in linear and cyclic configurations. The results show a marked increase in overall gas emissions throughout the experiment, with a notable correlation with temperature. While we did not directly measure the composition of the secondary particles formed in this study, the mass spectrometry results suggest that they likely include oxidized organic compounds.

## Conclusions

Our study reports the first investigation of secondary particulate matter formation from gaseous brake wear emissions. Due to the decrease in exhaust emissions, non-exhaust emission sources such as brake wear are becoming an increasingly important contributor to ambient PM. However, there is currently a lack of knowledge regarding the importance of gaseous emissions from brake wear and their fate in the atmosphere. The present study is part of a wider project investigating the gaseous emissions from brake wear and their fate in the atmosphere. The specific aim of this particular study was to test whether the photochemical ageing of gaseous emissions from the braking process might contribute to the burden of secondary PM. We conclusively show first evidence that brake wear emissions can form secondary PM. Such secondary PM from gaseous brake wear emissions might additionally contribute to the environmental impact of non-exhaust emissions from brakes. However, emissions factors for organic gaseous emissions from brake wear as well as the particle formation yields for different types of brakes are not known at this point, making it difficult to quantify this impact. A recent study of ceramic and semi-metallic brake pads showed strongly increased VOC emissions at heavy vs. light braking conditions^24^, while an earlier study of a low-metallic pad also highlighted the importance of braking history^23^. Quantifying the emissions and the particle formation yields for a variety of different brake pads and braking conditions would therefore be an important next step in this area of research. Such future studies should also investigate the impact of environmental factors such as temperature on the primary VOC emission. In addition, it would be important to confirm the results of this study via disc brake test stand and car field tests. Further studies should also investigate potential interactions with exhaust emissions.

The impact of airborne particles on health and climate also depends upon their physical and chemical properties. For example, a study comparing the biological effects of different brake pad formulations found that low-metallic pads did not lead to any biological response in the used in vitro model, whereas particles generated from non-asbestos organic (NAO) pads triggered inflammation and cytotoxicity^26^. This difference was attributed to the different compositions of the generated particles. The composition and toxicological properties of secondary brake wear particles are so far still completely unknown, although our results indicate that at least some of the secondary particle mass is likely comprised of oxidised organic compounds. Future studies should therefore ideally include measurements of particle composition as well as toxicological endpoints. In the meantime, the present study indicates that the photochemical ageing of the gaseous emission from brake wear is important to further study and should be considered in future regulations on non-exhaust emissions from road traffic.

## Materials and methods

### Generation of brake wear emissions

Brake wear emissions were generated using a pin-on-disc tribometer. The tribometer setup is the same as in a previous study^12^, with the exception that dried synthetic air (relative humidity < 5%) from a zero air generator (Sabio, model 1001) was used instead of filtered room air to ventilate the tribometer chamber at a flow of 7.5 L min^−1^. The air inside the chamber gets well mixed due to the geometry of the pin-on-disc machine body. Wear emissions are generated through contact between the dead-weight-loaded pin sample and the horizontal rotating disc sample. The emissions are sampled at the air outlet of the chamber. A schematic of the test stand is provided in Fig. S2. Pin-on-disc tribometers are relatively cheap to run, but simplify the real system. For run-in conditions, promising correlations for airborne particle size distributions and morphology have been demonstrated between the pin-on-disc test stand, disc brake test stand and car field tests^27^. For the more transient conditions, when the temperature transition to more ultrafine particles occurs, an increase in ultrafine emissions above the transition temperature is verified for both inertia dynamometer benches and pin-on-disc tribometers^28^. In this study, a cast iron vehicle brake disc was used as the rotating disc, while the pin was manufactured from a Non-Asbestos Organic (NAO) brake pad. This NAO pad material has been a reference NAO material in the European project REBRAKE and chemical specifications of the tested pad and rotor material can be found in Alemani et al.^12^. All experiments were performed at a contact pressure of 0.9 MPa and a sliding speed of 4 m s^−1^. This test condition is among those that can occur in urban and local road driving^8^.

### Ageing of gaseous emissions and measurement of particle formation

**Fig. 3:**
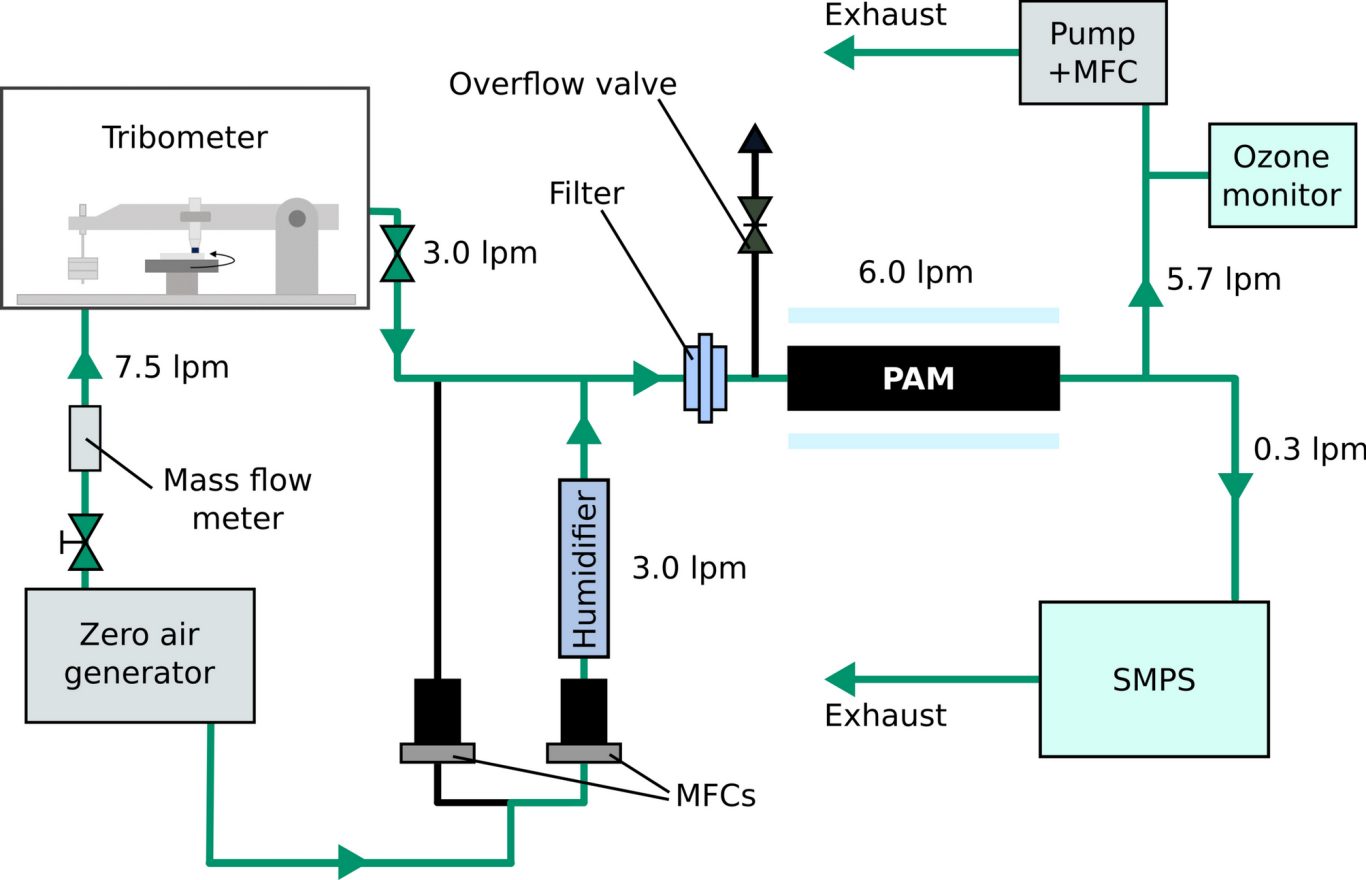
Experimental setup. During the ageing experiment, simulated brake wear emissions are generated in the tribometer. The emissions are then diluted 1:1 with humidified zero air, and filtered using a HEPA filter before entering the PAM oxidation flow reactor. The PAM reactor generates OH radicals to simulate atmospheric ageing. Formation of new PM is measured using an SMPS. In cleaning mode, the second MFC is set to a flow of 3.0 L min^−1^ dry zero air which is mixed with humidified air to provide the same flow rate and humidity into the PAM as during experiments.

A Potential Aerosol Mass (PAM) flow reactor (Aerodyne Research Inc, Billerica, MA, USA) was used to simulate photooxidative ageing of the gaseous brake wear emissions. It has been shown that SOA particles generated using a PAM reactor have similar chemical compositions to those generated in environmental chambers^29^.The PAM reactor was run in OFR185 mode, in which hydroxyl radicals (·OH) are generated through photolysis of both ozone (O_3_) and water^30^. The two low-pressure mercury lamps were attenuated 75% to reduce the equivalent atmospheric ageing time. A particle filter (9933-05-AQ, Parker, OH, USA, 99.9999 + % efficiency at 0.01µm) was connected at the inlet of the PAM reactor to remove any primary particles. The total airflow through the 13.3 L reactor was 6 L min^−1^, resulting in a residence time of 133 s. Three L min^−1^ of the total flow were directly sampled from the tribometer, while the remaining 3 L min^−1^ consisted of humidified synthetic air. The O_3_ concentration at the reactor outlet was measured using an O_3_ monitor (Model 205, 2B Technologies, Boulder; CO, USA), and the number size distributions of newly formed particles were monitored using a Scanning Mobility Particle Sizer (SMPS) consisting of a differential mobility analyser (DMA, model 3071A, TSI GmbH, Aachen, Germany) and a condensation particle counter (CPC, model 3776, TSI GmbH, Aachen, Germany). The size range in SMPS was set from 10 to 400 nm with a scan time of four minutes. The total flow through the PAM reactor was maintained by a pump in combination with a mass flow controller (MFC). Before each experiment, all flows were checked using a flow calibrator (Gilibrator 2, Sensidyne, St. Peterburg, FL, USA). A schematic of the experimental setup is shown in Fig. 3.

Experiments investigating the ageing of gaseous brake wear emissions were conducted for two different equivalent ageing times: (I) 7 days equivalent ageing and (II) 9 days equivalent ageing, with two replicates per equivalent age. The PAM reactor settings were (I) lamp voltage = 1.6 V (average irradiance = 0.02 μW cm^−2^), O_3_ = 781–926 ppb, and integrated OH exposure = 8.9 × 10^11^ molecules s cm^−3^), (II) lamp voltage = 2.0 V (average irradiance = 0.54 μW cm^−2^), O_3_ = 1848–2000 ppb, integrated OH exposure = 1.3 × 10^12^ molecules s cm^−3^. Temperature (T) and relative humidity (RH) were kept stable in the ranges of 28–30°C and 40–42% respectively for all experiments. (Fig. S3).

In addition to the experiments above, a control experiment was conducted at 2.0 V where the tribometer was run without a brake pin. Integrated OH exposure was determined in a set of separate experiments by measuring the decay of a known concentration of SO_2_^31^. The equivalent age was calculated assuming a daily average OH concentration of 1.5 × 10^6^ molecules cm^−3^ and using k_OH+SO2_ = 1.10 × 10^−12^ cm^3^ molecule^−1^ s^−1^ (NIST Chemical Kinetics Database, https://kinetics.nist.gov/kinetics/). The PAM reactor was cleaned before each experiment using UV-generated OH radicals in a flow of clean air. Before the start of each wear emission ageing experiment, the particle formation from background air under OH exposure was measured while the tribometer was off. The tribometer was then turned on to generate wear emissions, marking the start of the main experiment (time = 0).

### Measurement of freshly emitted gaseous organics

Separate experiments were performed to determine the chemical composition of the fresh gaseous emissions from brake wear using a Proton-Transfer-Reaction Mass Spectrometer (PTR-MS, IONICON FUSION). The chemical ionization was performed by hydronium ions (H_3_O^+^). The resolution of the instrument was about 7000 allowing for the discrimination of isotopes. Spectra were averaged and recorded every second up to *m/z* = 770, but detection was particularly sensitive to masses between 30 and 100 atomic mass units (amu). In these experiments, the same tribometer settings were used as for the ageing experiments, but the air was directly sampled from the tribometer chamber without photochemical ageing. Detailed results on the gas-phase measurements including the testing of further materials and parameters will be presented elsewhere.

### Safety considerations

Oxidation flow reactors can generate high concentration of gas phase oxidants such as ozone which can be harmful to human health. The flow system should therefore be thoroughly leak tested before operation and the exhaust needs to be vented appropriately. Use of an ozone alarm is recommended.

## Supplementary Information

Supplementary Information.

## Data Availability

The datasets supporting this study’s findings are available from the corresponding author upon reasonable request.
