# Supplementary material for: Gaseous emissions from brake wear can form secondary particulate matter
Source: Sci Rep. 2024 Oct 6;14:23253. doi: 10.1038/s41598-024-74378-5 (PMC11456579; doi:10.1038/s41598-024-74378-5)
Supplement: Supplementary file 1 — Supplementary Information. [file 41598_2024_74378_MOESM1_ESM.docx]

Supplementary information for

Gaseous emissions from brake wear can form secondary particulate matter

Anil Patel^1,2,a^, Sneha Aggarwal^1,2^, Lucas Bard^3^, Olivier Durif^4^, Micol Introna^1^, Ana Teresa Juárez-Facio^1^, Minghui Tu^3^, Karine Elihn^1^, Barbara Nozière^4^, Ulf Olofsson^3^, Sarah S. Steimer^1,2*^

^1^Department of Environmental Science, Stockholm University, Stockholm, 11418, Sweden

^2^ Bolin Centre for Climate Research, Stockholm, 11418, Sweden

^3^Department of Machine Design, KTH Royal Institute of Technology, 10044, Stockholm, Sweden

^4^ Department of Chemistry, KTH Royal Institute of Technology, 10044, Stockholm, Sweden

^a^now at: Department of Atmospheric and Oceanic Sciences, University of California at Los Angeles, Los Angeles, CA 90095-1565, USA

*Corresponding author. Email address: sarah.steimer@aces.su.se

*This supplement contains three figures;*

**Figure S1:** Development of the number size distribution in the PAM reactor over the tribometer running time

**Figure S2:** A schematic drawing of the tribometer

**Figure S3:** The PAM reactor settings, e.g., Relative Humidity (% RH), Temperature (ºC), and Ozone (ppb), over the tribometer running time


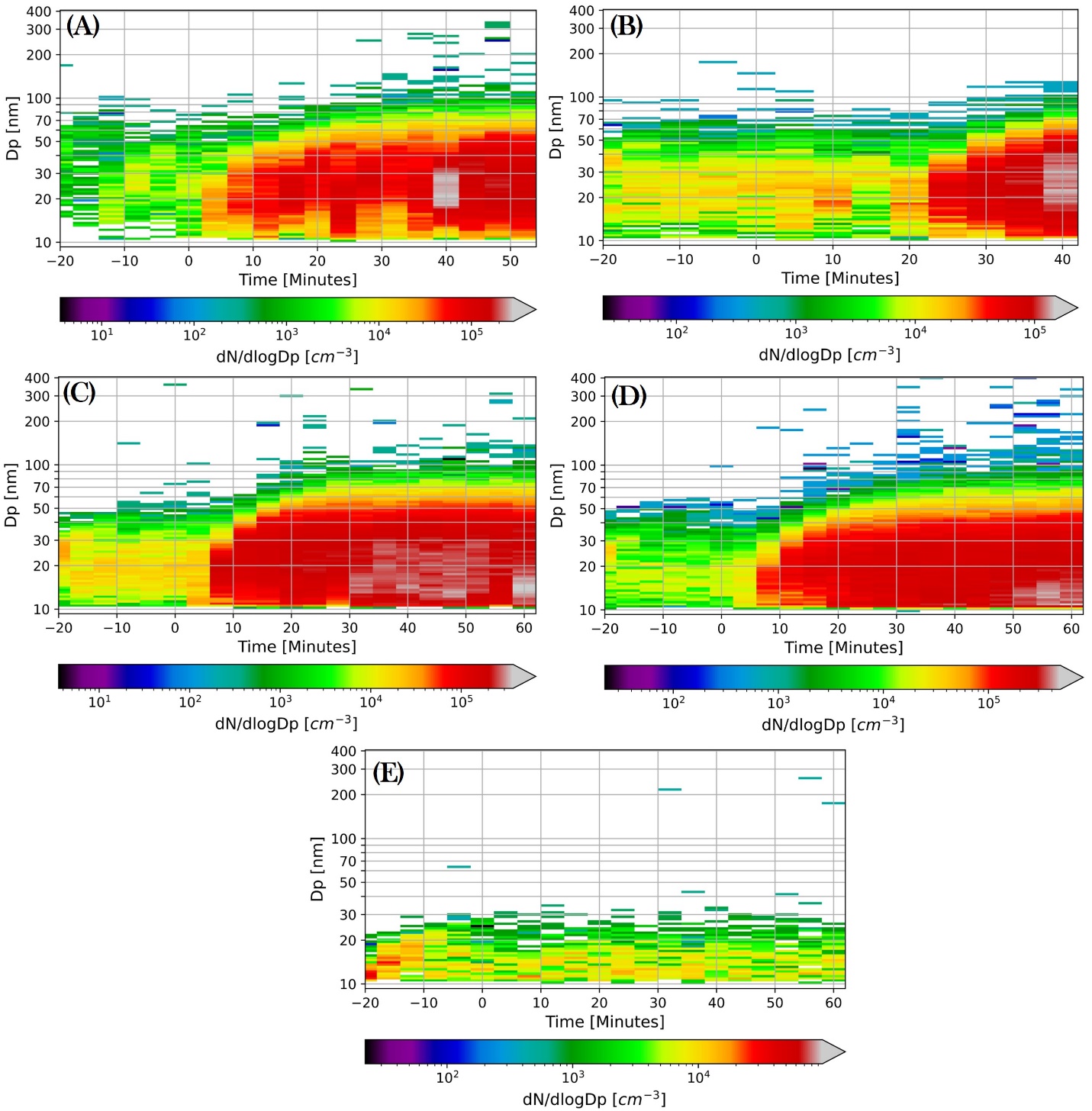


**Figure S1:** Development of the number size distribution in the PAM reactor over the tribometer running time for experiments obtained with (A) 7 days and (C) 9 days equivalent aging. B and D show the replicate of each equivalent aging time (7 respective 9 days). E shows the control experiment where the tribometer was run without a brake pin. Time 0 min marks the time when the tribometer was turned on (i.e. start of the generation of wear emissions for all experiments).


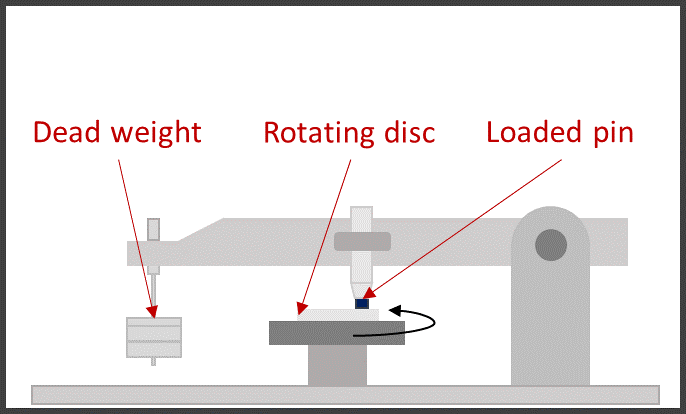


**Figure S2**: Schematic drawing of the pin-on-disc tribometer. Wear emissions are generated through contact between the dead-weight-loaded pin sample and the horizontal rotating disc sample. The dead-weight can be adjusted to generate different contact pressures. The tribometer machine body is enclosed in a chamber.


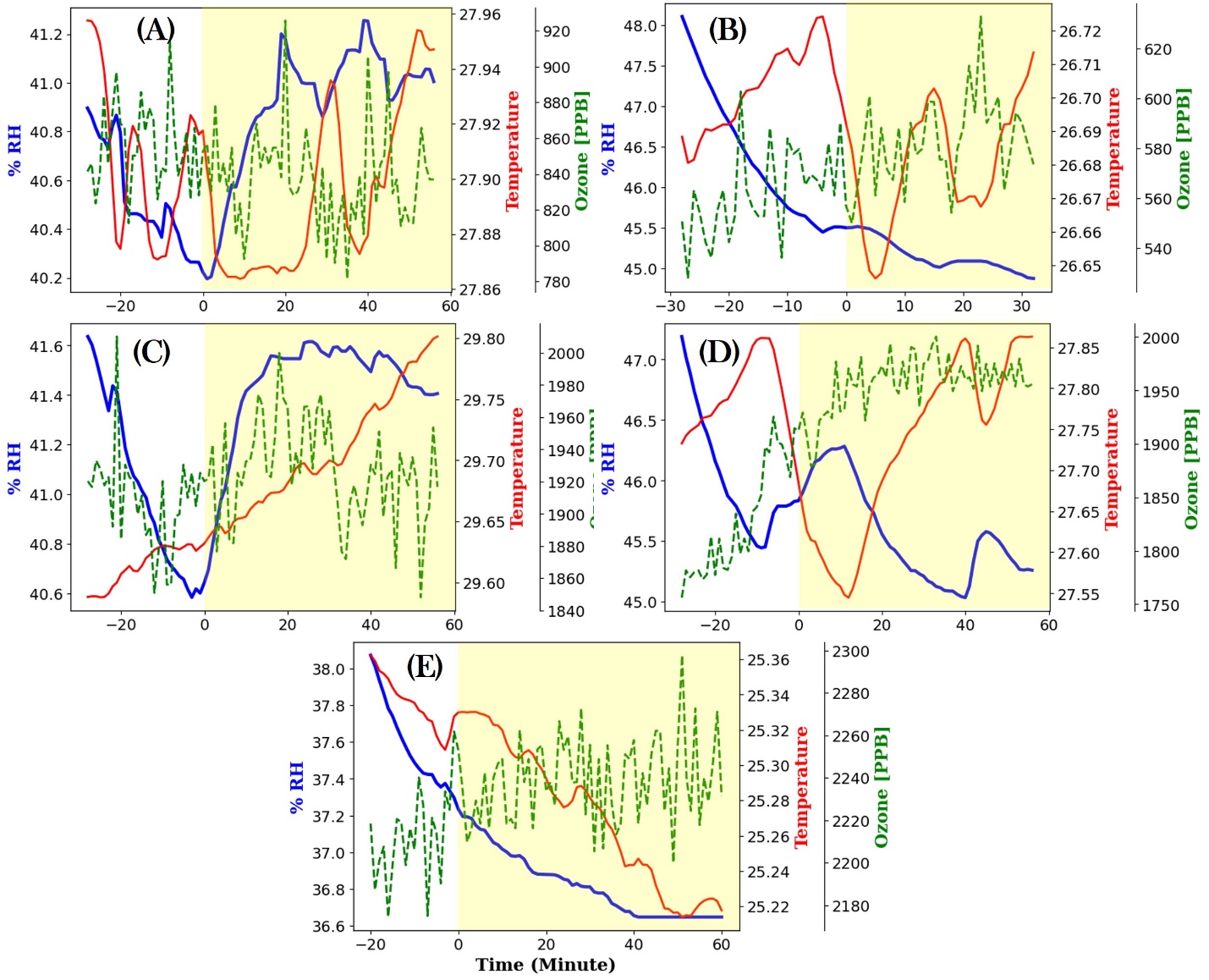


**Figure S3:** The PAM reactor settings, e.g., Relative Humidity (% RH, solid blue line), Temperature (ºC, solid red line), and Ozone (ppb, dashed green line), over the tribometer running time for experiments obtained with (A) 7 days and (C) 9 days equivalent aging. B and D show the replicate at each equivalent aging time (7 respective 9 days). E shows the control experiment where the tribometer was run without a brake pin. The yellow shading indicates the time periods during which the tribometer was running (i.e. wear emissions were generated).
